# Supplementary material for: All-at-once RNA folding with 3D motif prediction framed by evolutionary information
Source: Nat Methods. 2025 Oct 3;22(10):2094–106. doi: 10.1038/s41592-025-02833-w (PMC12510878; doi:10.1038/s41592-025-02833-w)
Supplement: Supplementary file 3 — Source code to run CaCoFold-R3D. Data and results. [file 41592_2025_2833_MOESM3_ESM.gz › supplemental_material/results/CaCoFold-R3D/CaCoFold-R3D_Rfam_vshuffle/RF01957_NEAT1_3.R2R.sto.pdf]

RF01957_NEAT1_3

5′ U A G U G U U C A U C A U G G C G A C A G A U G G C A C C A G G A G A A A U G G A G C C C C U G G C C A G U G U G A G U C R U A G C A G U G C A G G A G G G G A G A C C C U G R A G G A G A G A G C C C G C C U A A R U U G A U G Y C U G C A G A Y U G A A U U U C C A G A G G C U U A G G A G R G G A A G Y U C U C C A
